# Discontinuous Highly Aligned Carbon Fibre Tapes Combined with Bio-Based Polyamide 11

**DOI:** 10.3390/polym18151878

**Published:** 2026-07-30

**Authors:** Christian Brauner, Florian Givel, Julian Kupski, Lucian Zweifel, Mohammad Hajikazemi, Miriam Preinfalck, Stephan Baz, Götz T. Gresser

**Affiliations:** 1 Institute of Polymer Engineering, FHNW University of Applied Sciences and Arts Northwestern Switzerland, Klosterzelgstrasse 2, 5210 Windisch, Switzerland; florian.givel@fhnw.ch (F.G.); julian.kupski@fhnw.ch (J.K.); lucian.zweifel@fhnw.ch (L.Z.); mohammad.hajikazemi@fhnw.ch (M.H.); 2 Deutsche Institute für Textil- und Faserforschung (DITF), Körschtalstraße 26, 73770 Denkendorf, Germany; miriam.preinfalck@ditf.de (M.P.); stephan.baz@ditf.de (S.B.); goetz.gresser@ditf.de (G.T.G.)

**Keywords:** bio-based polyamide 11, recycled carbon fibre, aligned discontinuous fibres, composite tape, additive manufacturing, 3D printing, sustainable composites

## Abstract

The increasing availability of recycled carbon fibres (rCFs) from manufacturing waste and end-of-life composite structures offers new opportunities for sustainable, high-performance composites. However, the discontinuous nature of recycled fibres requires efficient alignment and consolidation. In this study, highly aligned discontinuous carbon fibre tapes were manufactured from recycled carbon staple fibres and bio-based polyamide 11 (PA11) fibres using a textile-based processing route and compression moulding. Two material systems containing nominal fibre mass fractions of 50 wt.% and 70 wt.% rCF were investigated. The resulting laminates were characterised in terms of fibre volume content (FVC), fibre orientation distribution, and mechanical performance. FVC of up to 56.72 vol.% were achieved for the 70 wt.% rCF material. Tensile testing revealed a significant increase in stiffness and strength with increasing fibre content, reaching values of 48.1 GPa and 866 MPa. The longitudinal compression modulus and the in-plane shear modulus showed similar trends, while the transverse tensile strength and compressive strength remained strongly influenced by local defects and fibre–matrix interactions. A 2D mesoscopic image analysis demonstrated a pronounced preferential fibre orientation, with approximately 69% of the analysed fibre regions aligned within ±10° of the dominant fibre direction. The derived alignment coefficient η_0_ correlated well with the tensile modulus, confirming the strong influence of mesoscopic fibre architecture on mechanical performance. The results demonstrate that the combination of bio-based PA11 and oriented rCF, enabled through textile-based alignment technologies, provides a promising pathway towards sustainable lightweight composite structures.

## 1. Introduction

The growing demand for sustainable lightweight materials has driven the development of composite systems based on recycled reinforcements and bio-based polymer matrices. Polyamide 11 (PA11) is a bio-based engineering thermoplastic derived from castor oil and it offers mechanical properties comparable to polyamide 12 (PA12), making it a promising matrix material for sustainable thermoplastic composite applications. Recycled carbon fibres (rCFs) recovered from manufacturing waste or end-of-life composites retain a large proportion of the stiffness of virgin carbon fibres (vCFs) while requiring substantially lower material and energy inputs for their production. Their use in new composite materials therefore addresses both resource efficiency and waste reduction [1,2,3,4,5,6,7]. However, a key challenge associated with rCFs is their typically discontinuous nature and random orientation, which have historically limited their use to lower performance applications [8]. Misalignment and discontinuity of fibres lead to significantly reduced mechanical properties compared with continuous fibre composites [1,9].

To fully exploit the mechanical potential of rCFs, strategies to align and concentrate them in the load direction are essential. Aligned discontinuous fibre-reinforced composites can approach the performance of continuous fibre composites if a high degree of alignment and fibre volume fraction is achieved [8,10,11,12]. Recent advances in processing have demonstrated methods for re-aligning discontinuous recycled fibres into unidirectional preforms and tapes. For example, Detzel et al. [13] further processed recycled carbon staple fibre yarns into unidirectionally reinforced tapes by stretching and calendaring, resulting in fibre alignment improvements from 66% to 92% within ±10° and corresponding improvements in tensile stiffness and strength. Compared with previously reported alignment approaches for recycled carbon fibres, which are largely based on centrifugal deposition, air-laid processes or short-fibre carding and are typically limited to fibre lengths below 20–25 mm, the textile-based sliver process employed in this study [11,12] is capable of processing and aligning considerably longer rCF staples of approximately 80 mm. This retained fibre length is expected to be beneficial for load transfer and mechanical performance. Such studies show that, with appropriate fibre handling and processing, recycled fibres can be formed into semi-finished products (tapes, yarns) suitable for structural composite applications.

Continuous fibre thermoplastic processing technologies such as additive manufacturing (AM) [14], automated tape laying (ATL) [15], and automated fibre placement (AFP) [16] could benefit significantly from sustainable feedstocks. In particular, fused filament fabrication and related AM processes have been adapted to utilise continuous carbon fibre/thermoplastic tapes, enabling the production of lightweight components with high specific strength and stiffness [17]. To date, large scale applications of these technologies rely predominantly on virgin carbon fibre combined with polyamide 12 matrix systems (vCF/PA12) [18], presenting a significant environmental footprint. The integration of rCFs into tape-based thermoplastic processing routes could therefore provide a pathway towards more resource-efficient lightweight structures. Internal life cycle assessment (LCA) studies indicated that the greenhouse gas emissions associated with rCF/PA11 components may be as low as 10–30% of those of equivalent aluminium parts, depending on the application and assumptions considered. Consequently, the combination of bio-based PA11 and rCF as a tape-based feedstock for additive manufacturing represents a promising material system for sustainable lightweight construction.

This study first presents the development of a PA11/rCF composite tape produced via a textile-based processing route and tailored for automated deposition processes such as continuous fibre AM and AFP. The work focuses on achieving high fibre contents (50–70 wt.% rCF, corresponding to ~35–57 vol.%) while maintaining good fibre alignment and sufficient tape quality for subsequent processing. The manufacturing route includes melt spinning of PA11 matrix fibres, fibre opening and alignment, tape formation, post-consolidation, and slitting to final tape dimensions. Second, the resulting laminates are characterised in terms of fibre volume content (FVC), fibre orientation distribution, microstructure, and mechanical performance under tensile (0° and 90°), compressive and shear loading. Particular attention is given to the influence of fibre orientation and processing-induced defects on the mechanical properties. Key processing challenges, including fibre impregnation, porosity reduction, and consolidation quality, are discussed. The objective is to assess the viability of PA11/rCF tapes as sustainable alternatives to conventional virgin carbon fibre (vCF)/PA12 tapes for continuous fibre additive manufacturing, ATL or AFP applications [12]. By benchmarking the material properties against those of commercially available vCF/PA12 tapes, the current performance gaps and potential pathways for further improvements are identified.

## 2. Materials and Methods

### 2.1. Fibre and Matrix Materials

A commercial bio-based PA11 (Rilsan^®^ PA11, Arkema SA, Colombes, France) was used as the matrix polymer. PA11 is derived from castor oil and is predominantly based on renewable feedstock. The material has a melting temperature of ~187 °C and was supplied in pellet form. Prior to processing, the pellets were dried at 80 °C to minimise moisture-induced degradation.

The recycled carbon fibres (rCF) originating from T700SC-12K-50C production offcuts (Toray Industries, Inc., Tokyo, Japan) were used as reinforcement. T700SC is a standard-modulus, high-strength carbon fibre grade commonly used in structural composite applications. The fibres had an average fibre length of approximately 50 mm and an average fibre diameter of 7 μm.

### 2.2. Tape Fabrication Process

**Figure 1:**
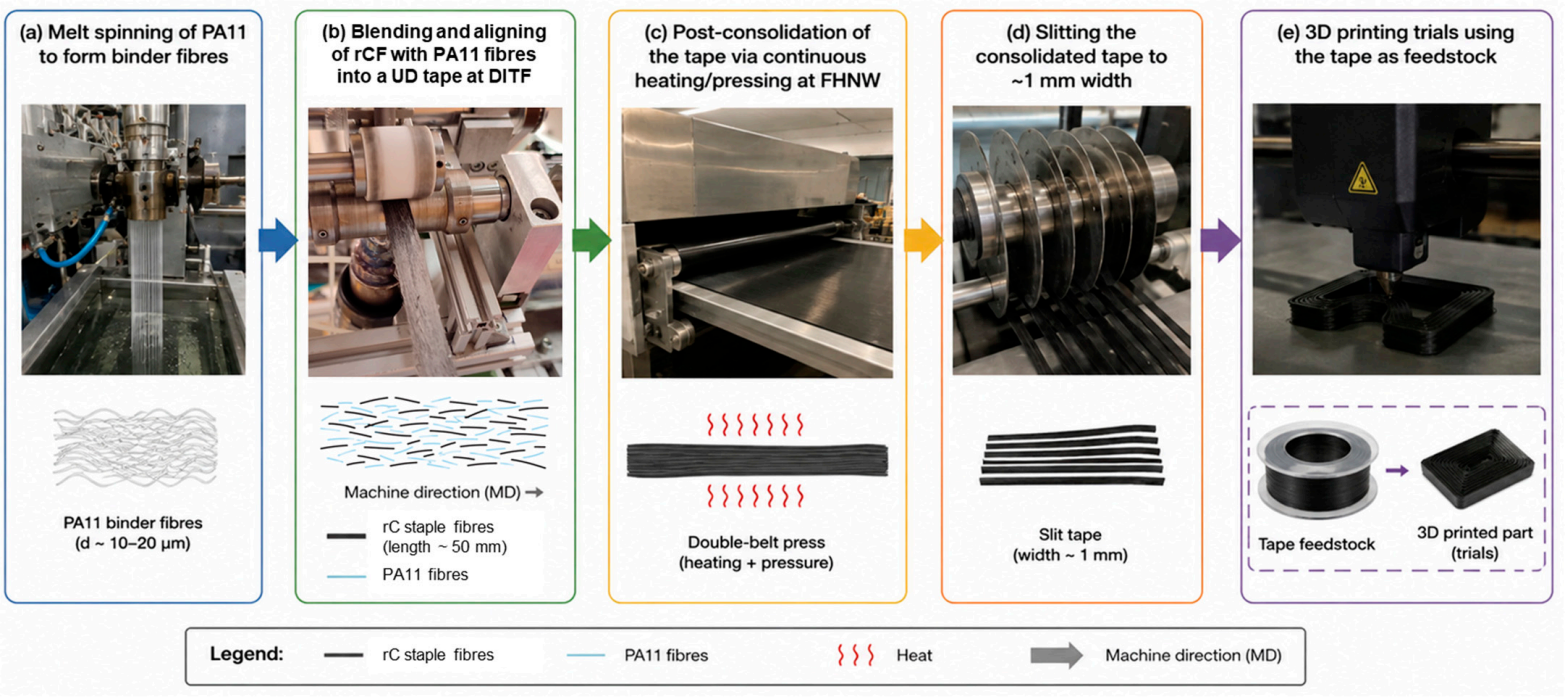
Schematic of the PA11/rCF tape production process: (**a**) melt spinning of PA11 fibres; (**b**) opening, bending and alignment of rCF and PA11 fibres into a unidirectional tape; (**c**) post-consolidation of the tape using a continuous double-belt press; (**d**) slitting the consolidated tape to ~1 mm width; and (**e**) 3D printing trials using the tape as feedstock.

The manufacturing of PA11/rCF composite tape involved several sequential steps carried out at specialised facilities, as illustrated in Figure 1. The overall strategy was to use PA11 in fibrous form to support fibre blending and alignment, followed by thermal consolidation to melt the PA11 and bind the aligned rCF structure into a tape. The target fibre mass fractions of 50–70 wt.% were achieved by adjusting the ratio of rCF to PA11 fibres.

Melt spinning of PA11 fibres: PA11 pellets were melt-spun to create continuous PA11 microfibres, which served as thermoplastic binder fibres during the tape manufacture. Melt spinning was performed using a laboratory-scale fibre spinning line (Fourné Maschinenbau GmbH, Alfter-Impekoven, Germany), with the PA11 being extruded through a spinneret at ~250 °C. Key process parameters, including the extruder temperature profile, take-up speed, and quench conditions, were adjusted to obtain uniform filament diameters of ~20–40 µm. The spun PA11 fibres were collected as tows and subsequently cut to staple lengths of 60 mm to approximately match the average rCF length after carding, thereby ensuring comparable fibre lengths for the subsequent drafting and alignment process.

Fibre opening and carding: The primary tape manufacturing step was carried out at DITF (Denkendorf, Germany) using a textile processing approach for staple fibres [11]. First, the rCF and PA11 fibres were opened and blended using an opener system by DILO Machines GmbH (Eberbach, Germany). The ratio of rCF to PA11 fibres fed was adjusted to obtain target fibre mass fractions of 50 wt.% and 70 wt.% rCF. The blended fibre material was subsequently processed using a carding line from DILO Machines GmbH consisting of a feeding chute K11, a KC11 compact card and a sliver coiler RS600 with extraction unit RSSU-L from Rosink GmbH + Co. KG Maschinenfabrik (Nordhorn, Germany), in which the rCF bundles and PA11 fibres were gently separated, homogeneously blended and parallelized to promote fibre alignment and finally extracted from the machine in the form of a fibre sliver.

**Figure 2:**
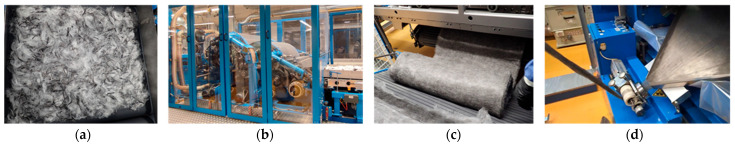
Two-stage carding process: (**a**) feeding of the pre-opened PA11/rCF fibre blend; (**b**) roller carding process with KC11; (**c**) resulting dry nonwoven used for re-feeding into the carding line; and (**d**) resulting PA11/rCF fibre sliver at the output of the RSSU-L extraction unit of the sliver coiler RS600.

During preliminary trials, the uncrimped rCF/PA11 fibre blend exhibited insufficient cohesion during carding, resulting in poor card web uniformity. To improve process stability and web homogeneity, a two-stage carding approach was developed. In the first stage, the fibre web was processed through the cross-lapper DLAK11 from DILO Machines GmbH of the carding line to produce an unbonded dry nonwoven with approximately 10 stacked layers, which was subsequently rolled up (Figure 2a–c). In the second stage, this dry nonwoven was re-fed into the carding line, after which the resulting fibre web was condensed into a fibre sliver of approximately 4 ktex within the sliver coiler and stored in cans for subsequent tape formation (Figure 2d).

Fibre alignment and tape formation: Initially, tape formation was performed in a continuous process combining drafting and consolidation by using a prototype machine designed by the DITF [19]. The aligned rCF/PA11 fibre slivers were drafted to reduce the sliver thickness and increase fibre alignment. Simultaneously, the PA11 fibres were heated above their melting temperature (~190–210 °C) to provide thermal bonding of the fibre structure. The material was subsequently calendared into a thin tape and cooled to stabilise the tape geometry.

Following preliminary processing trials, the drafting and consolidation stages were separated to improve process control. In the revised process, the fibre sliver was first drafted in a hot-air zone on the DITF prototype machine. Total draft ratios between 2 and 5 were applied to reduce the sliver thickness and further increase fibre alignment. This enabled partial melting of the PA11 fibres, providing sufficient thermal bonding for subsequent handling while maintaining the aligned fibre architecture. The process was operated at line speeds between approximately 0.5 and 2 m/min. The pre-stabilised tape was then subjected to a separate consolidation step.

Post-consolidation (secondary pressing): To improve tape quality, especially impregnation and surface matrix coverage, a post-consolidation step was applied to selected tapes. A commercial continuous band sealer was modified and extended to operate as a laboratory-scale double-belt consolidation system. The system was equipped with tape guiding, process monitoring, and active cooling to enable continuous thermal consolidation under controlled conditions.

The tapes were passed through the heated belt section at ~210 °C, where they were consolidated between continuously moving belts under pressure, followed by controlled cooling. The post-consolidation step was intended to improve matrix distribution and overall tape consolidation prior to subsequent processing and characterisation.

It should be noted that the post-consolidation step does not influence fibre orientation, which is established during the preceding textile alignment and drafting stages, but is solely intended to improve impregnation and reduce porosity.

Slitting: The consolidated tapes, with widths of approx. 12 mm, were slit into narrower tapes suitable for subsequent additive manufacturing and compression moulding trials. For additive manufacturing, a target tape width of 1 mm was selected to match the tape guiding system of the printing device. For compression moulding, wider tapes of approximately 10 mm were used for manual layup. Slitting was performed using a razor slitter integrated into a roll-to-roll web-processing line. Process parameters, including blade position, blade sharpness, and tape tension, were adjusted to obtain tape strips with the desired dimensions.

For additive manufacturing trials, only tape sections meeting the dimensional requirements of the printing system were selected. Sections exhibiting excessive thickness variation were removed prior to processing. The selected tape sections had thicknesses of approximately 0.20 mm, compared with approximately 0.16 mm for the commercial carbon fibre/PA12 reference tape used in the printing system [17].

### 2.3. Compression Moulding

Consolidation of the PA11/rCF tapes into flat composite plates was performed using a programmable double-platen hydraulic press (Vogt Labormaschinen GmbH, Berlin, Germany).

The consolidation cycle comprised six main steps:

1. Initial seating: the mould was closed under low pressure (~2 bar) at room temperature to seat the preform.
2. Heating phase: the mould was heated to 210 °C under low pressure (~2 bar) and held for ~15 min.
3. Pressure ramp: the pressure was gradually increased to the target consolidation level, ranging from 20 to 100 bar depending on the material system and processing condition.
4. Consolidation hold: the target pressure and temperature were maintained for 30 min to ensure full melt and pressure-assisted impregnation.
5. Controlled cooling: the part was cooled to 150 °C under constant pressure using an integrated water-cooled system.
6. Final release: after reaching 150 °C, the pressure was released and the mould was opened at room temperature (~25 °C).

Both material systems (50 wt.% and 70 wt.% rCF) were consolidated using this procedure. For the 70 wt.% rCF/PA11 tapes, additional consolidation iterations were conducted at increasing pressure levels up to 100 bar to improve impregnation and eliminate voids. Plate thicknesses were monitored after each cycle to assess uniformity and quality.

### 2.4. Mechanical Testing

Tensile tests were conducted in accordance with ISO 527-5:2021 [20], which specifies conditions for testing unidirectional fibre-reinforced plastic composites. The specimens were prepared as type A (for 0° and 90° fibre orientations) with a standard gauge length of 60 mm and end tabs bonded using epoxy. Testing was performed using a universal testing machine (ZwickRoell GmbH & Co. KG, Ulm, Germany) equipped with a 100 kN load cell. Strain was measured using bonded strain gauges aligned parallel and perpendicular to the fibre direction. Constant crosshead displacement rates of 2 mm/min (UD 0°) and 1 mm/min (UD 90°) were applied.

Compression tests were performed in accordance with ISO 14126:2023 [21], which determines the compressive properties of fibre-reinforced plastics in-plane. The method employed was the combined loading compression (CLC) fixture setup with a gauge length of 10 mm and fitted with bonded end tabs. The specimen ends were polished to ensure uniform load introduction. Strain measurement used biaxial strain gauges, and the crosshead speed was set to 1 mm/min.

In-plane shear tests were carried out in accordance with ISO 14129:1997 [22] using the ±45° tensile test method. Rectangular specimens were cut from laminates with fibre orientation at ±45° to the loading axis. Tests were conducted at a constant displacement rate of 2 mm/min. The shear stress–strain response was calculated using the laminate theory assumptions for symmetric balanced ±45° layups. The total shear strain was derived from both the axial and transverse strains, which were measured using biaxial bonded strain gauges.

### 2.5. Image Analysis

To quantify the fibre orientation distribution (FOD) within the laminates, a two-dimensional mesoscopic image analysis approach, previously described in [11], was employed. The structure tensor methodology was implemented using Python (v3.8.12) and included three main steps: (1) image acquisition, (2) computation of the FOD, and (3) derivation and visualisation of the alignment coefficient $\eta_{0}$ in a binned plot. The alignment coefficient is a scalar parameter that represents the degree of fibre alignment along a specific direction and is defined as follows:

(1) $$\eta_{0}=\sum \beta_{i}\cos^{4}(\theta_{i}),$$

where $\beta_{i}$ is the frequency of the related fibre orientation angle $\theta_{i}$.

Images of the individual tape layers were taken prior to laminate consolidation using a Nikon D810 DSLR camera (Nikon Corporation, Tokyo, Japan) equipped with a SIGMA 50 mm f/1.4 DG HSM lens (SIGMA Corporation, Kawasaki, Japan) and a Walimex circular polarisation filter (Walimex, Burgheim, Germany) to reduce glare and reflection artefacts from the carbon fibres. This setup produced high-resolution 5759 × 2879 pixel images at 300 dpi and 24-bit depth, yielding an effective resolution of approximately 0.24 pixels per fibre. A checkerboard calibration procedure based on OpenCV libraries was used to correct geometric distortion and in-plane effects.

Each image was processed using an automated Python script. The image was subdivided into smaller blocks (segments), serving as homogenisation domains. Fibre orientation analysis was performed on each block using the structure tensor approach as implemented in the Scikit library. This method, based on local gradients, is computationally efficient and robust against noise, making it suitable for complex fibre architectures. Orientation histograms were extracted from each segment, and the alignment coefficient $\eta_{0}$ was computed based on the local probability density function.

Finally, the segments were aggregated across the entire plate and virtually stacked, yielding median and standard deviation values for $\eta_{0}$ for each plate configuration [11].

### 2.6. Scanning Electron Microscopy

Fracture surfaces of selected mechanically tested specimens were analysed using scanning electron microscopy (SEM) to provide qualitative insight into failure mechanisms and support interpretation of mechanical behaviour. Images were captured in secondary electron mode at an accelerating voltage of 2.0 kV, a working distance of approximately 8.4 mm, a 30 µm aperture, and ×100 magnification. SEM observations were used to assess fracture surface morphology and microstructural features of the PA11/rCF laminates, including fibre exposure, pull-out, matrix coverage, voids, and indications of dry or insufficiently consolidated regions.

### 2.7. Determination of Fibre Volume Content (FVC)

The fibre mass content (FMC) of the PA11/rCF composite materials was determined using three different methods: thermogravimetric analysis (TGA), muffle furnace decomposition and chemical dissolution. The corresponding fibre volume contents (FVCs) were subsequently calculated from the measured FMC values.

TGA was performed to determine the fibre mass content (FMC) through thermal decomposition of the polymer matrix. The procedure involved two sequential heating steps: In the first step, samples were first heated from room temperature to 600 °C at a rate of 10 °C/min under a nitrogen atmosphere and held isothermally for 20 min. During this phase, the polymer matrix degraded, leaving behind a residual mass comprising carbon fibres and any remaining decomposition residue. In the second step, the atmosphere was switched to air atmosphere and the temperature was increased from 600 °C to 900 °C at a rate of 10 °C/min. This oxidative step served to burn off the remaining carbonaceous components.

As an additional thermal method, the FMC was determined through matrix decomposition in a muffle furnace in accordance with the principles of ISO 14127 [23]. This method enables the analysis of larger sample quantities and serves as an independent validation of the TGA-based measurements. Composite specimens with a mass of ~0.5–2 g were placed in pre-weighed ceramic crucibles and covered with perforated aluminium foil to prevent material loss while allowing gas exchange. The samples were heated to 550 °C and held for 2 h to ensure complete thermal decomposition of the PA11 matrix without significant oxidation of the rCF. During this process, the PA11 matrix thermally decomposed, leaving a residual mass primarily consisting of carbon fibres. After slowly cooling to room temperature, the remaining residue was weighed and used to determine the FMC.

Fibre mass content (FMC) was additionally determined through selective dissolution of the PA11 matrix. This method is based on the selective dissolution of the polymer matrix while preserving the carbon fibres, which are subsequently recovered by filtration, dried, and weighed. Composite specimens with a mass of approximately 0.5–2 g were cut from representative laminate regions and weighed prior to testing. Several solvent systems were evaluated for their ability to dissolve the PA11 matrix without affecting the carbon fibres. A mixture of formic acid and dichloromethane (DCM) in a volumetric ratio of 50:50 was found to provide complete dissolution of the PA11 matrix while maintaining the integrity of the carbon fibres. The formic acid used was reagent grade with a concentration of ≥98%. The composite specimens were immersed in 40 mL of the solvent mixture and continuously agitated using a magnetic stirrer until complete dissolution of the matrix was achieved, which typically required 2–8 h depending on specimen size and fibre content. The remaining carbon fibres were separated from the solution by vacuum filtration using a Büchner funnel and a pre-weighed Whatman GF/F glass microfibre filter. The retained fibres were subsequently rinsed several times with acetone to remove residual solvent and any salt-like dissolution by-products. The filter containing the recovered fibres was transferred to a pre-weighed Petri dish and dried at 60 °C for a minimum of 48 h until a constant mass was reached. After cooling to room temperature in a desiccator, the recovered fibre mass was determined gravimetrically.

The fibre mass content (FMC) was calculated from the ratio of the recovered fibre mass to the initial specimen mass according to

(2) $$FMC=\frac{m_{f}}{m_{0}},$$

where $m_{0}$ is the initial composite specimen mass [g] and $m_{f}$ is the recovered dry fibre mass [g]. The fibre volume content (FVC) was determined from the measured FMC according to

(3) $$FVC=\frac{FMC/\rho_{f}}{\left(FMC/\rho_{f})+((1-FMC)/\rho_{m}\right)},$$

where $\rho_{f}$ is the density of carbon fibres (1.80 g·cm^−3^) and $\rho_{m}$ is the density of PA11 matrix (1.03 g·cm^−3^).

## 3. Results

### 3.1. Effect of Post-Consolidation on Tape Quality

**Figure 3:**
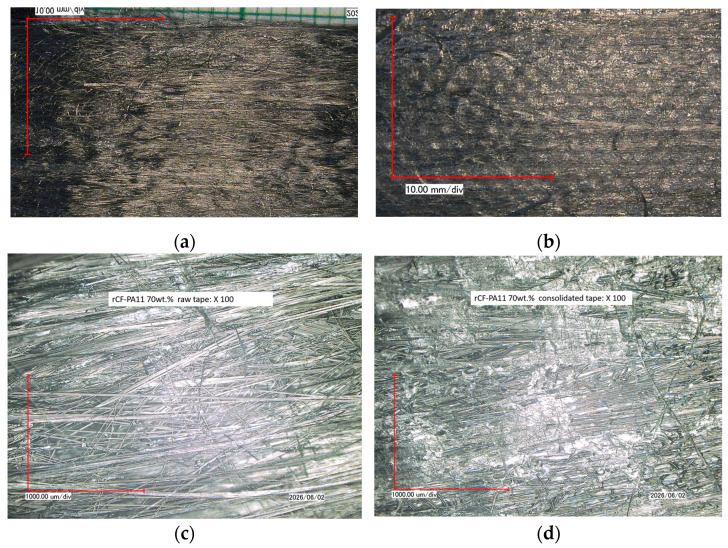
Surface micrographs of PA11/rCF tapes: (**a**) tape sample before post-consolidation; (**b**) tape sample after post-consolidation at 210 °C; (**c**,**d**) corresponding higher magnifications.

Figure 3 shows representative surface images of PA11/rCF tapes before and after post-consolidation at two different magnifications. Prior to post-consolidation (Figure 3a,c), individual PA11 fibres remained clearly visible within the tape structure, indicating that the matrix had not fully melted and redistributed during the initial tape-forming process. This indicates incomplete matrix redistribution and limited impregnation of the aligned carbon fibre network. Following post-consolidation at 210 °C (Figure 3b,d), the visibility of individual PA11 fibres was substantially reduced, indicating improved melting and redistribution of the matrix within the tape structure. A characteristic ribbed surface pattern introduced by the consolidation belts was visible after post-consolidation (Figure 3b), indicating effective pressure application during processing.

The additional consolidation treatment reduced thickness variation and promoted a more homogeneous distribution of the PA11 matrix, leading to a partial reduction in voids and dry regions. However, areas with insufficient initial matrix content remained only partially impregnated after reheating, indicating that full impregnation requires both sufficient matrix availability and adequate consolidation pressure. Another issue encountered was partial tape deconsolidation due to fibre spring back after exiting the heating zone when pressure was not sufficiently maintained during cooling. This resulted in elastic recovery of the tape structure and the formation of micro-voids during cooling. These observations indicated the importance of controlled cooling (e.g., water-cooled plates) and a fixed pressure application until complete solidification of the tape.

### 3.2. Microstructure of Compression-Moulded Laminates

The PA11/rCF tapes were successfully processed into flat laminate plates through compression moulding. The consolidation behaviour depended strongly on the nominal fibre mass content. The PA11/rCF 50 wt.% material could be consolidated using the initial pressing cycle, whereas the PA11/rCF 70 wt.% material resulted in incomplete impregnation after the first cycle, requiring additional consolidation steps at progressively increasing pressure. Increasing the consolidation pressure stepwise improved laminate quality and reduced the extent of locally dry regions. The best surface quality was obtained after consolidation at 210 °C and up to 100 bar. Nevertheless, the 70 wt.% material remained more sensitive to local impregnation deficiencies than the 50 wt.% material.

**Figure 4:**
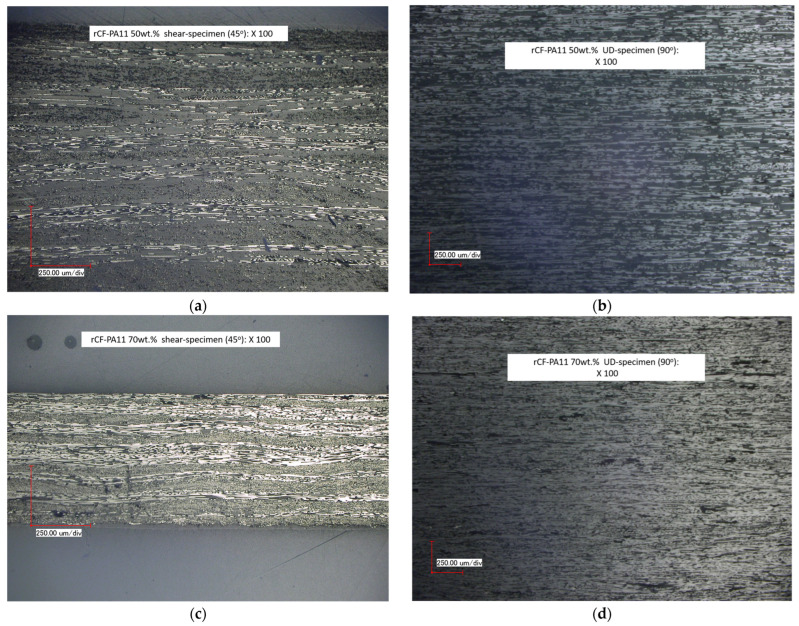
Cross-sectional micrographs of PA11/rCF laminates. (**a**,**b**) Samples with 50 wt.% fibre content; (**c**,**d**) samples with 70 wt.% fibre content.

Representative cross-sectional micrographs of the consolidated PA11/rCF laminates are shown in Figure 4. The images were obtained from specimens used for tensile and shear testing and illustrate the microstructure of both material systems (50 wt.% and 70 wt.% rCF).

The micrographs reveal a layered laminate architecture resulting from the stacking and consolidation of individual tapes. In the ±45° shear laminates (Figure 4a,c), fibre-rich layers and matrix-rich regions can be distinguished throughout the laminate thickness. The laminates containing 70 wt.% rCF (Figure 4c) exhibit a noticeably higher fibre packing density and reduced matrix-rich regions compared with the 50 wt.% rCF material (Figure 4a).

The cross-sections of the UD90 tensile laminates (Figure 4b,d) show a relatively homogeneous distribution of fibre cross-sections across the laminate thickness. The higher fibre content of the 70 wt.% material (Figure 4d) is clearly reflected by the increased density of fibre cross-sections compared with the 50 wt.% laminate (Figure 4b). Local heterogeneities, including fibre bundle accumulations and isolated dark regions, are visible in both material systems and indicate remaining microstructural inhomogeneities originating from the tape manufacturing and consolidation process.

Overall, the micrographs confirm the successful incorporation of the aligned PA11/rCF tapes into consolidated laminates while highlighting the strong influence of fibre content on laminate architecture, fibre packing density, and impregnation quality.

### 3.3. Tensile Test Results (ISO 527-5)

The tensile properties of the compression-moulded PA11/rCF laminates are summarised in Table 1. The results show a clear effect of fibre content on the longitudinal mechanical performance.

For the PA11/rCF 50 wt.% material, the longitudinal tensile modulus was 32.57 ± 0.55 GPa and the tensile strength reached 718.9 ± 34.2 MPa. Increasing the rCF content to 70 wt.% increased the longitudinal tensile modulus to 48.14 ± 4.55 GPa and the tensile strength to 866.2 ± 127.2 MPa. At the same time, the strain at failure decreased from 2.17 ± 0.11% to 1.78 ± 0.18%, indicating a stiffer and less ductile response at higher fibre content.

**Figure 5:**
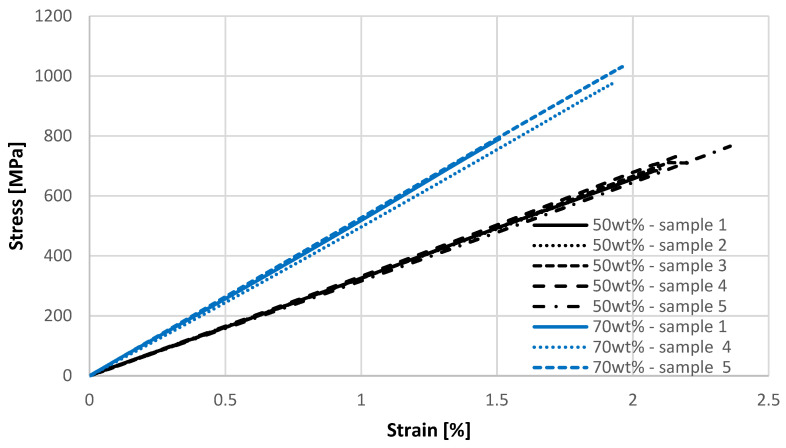
Longitudinal tensile stress–strain curves (UD 0°) of PA11/rCF 50 wt.% (*n* = 5) and 70 wt.% (*n* = 3) laminates.

Figure 5 shows the stress–strain behaviour of the UD 0° laminates. The substantial increase in modulus and strength demonstrates the effectiveness of increasing the fibre content in the aligned tape architecture. The improved longitudinal performance can be attributed to the higher fraction of load-bearing carbon fibres and the efficient transfer of load along the predominant fibre direction.

**Figure 6:**
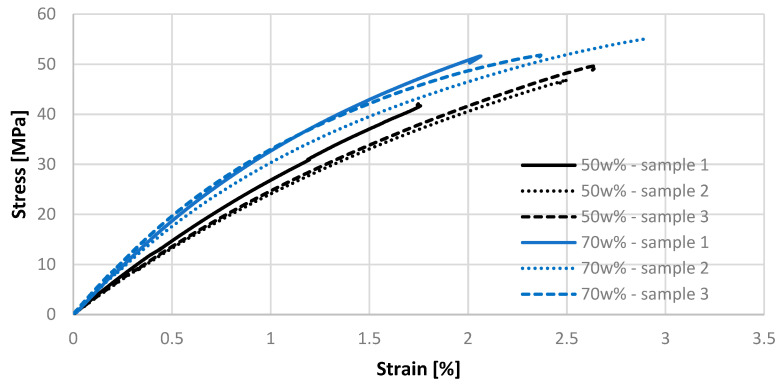
Transverse tensile stress–strain curves (UD 90°) of PA11/rCF 50 wt.% (*n* = 3) and 70 wt.% (*n* = 3) laminates.

The transverse tensile behaviour of the laminates is shown in Figure 6. In contrast to the longitudinal response, only a moderate increase in stiffness was observed with increasing fibre content, while the tensile strength remained nearly unchanged.

The transverse tensile properties were substantially lower than the corresponding longitudinal values. The UD 90° tensile modulus increased moderately from 2.92 ± 0.18 GPa for PA11/rCF 50 wt.% material to 3.91 ± 0.21 GPa for the PA11/rCF 70 wt.% material. In contrast, the transverse tensile strength remained nearly unchanged, with values of 50.6 ± 4.3 MPa and 52.9 ± 2.0 MPa, respectively.

This behaviour indicates that the transverse response is governed primarily by the PA11 matrix, fibre–matrix interface quality and local defects rather than by the axial stiffness of the carbon fibres. Consequently, increasing the fibre content has a much smaller effect on the transverse properties than on the longitudinal properties.

### 3.4. Compression Test Results (ISO 14126)

The compression test results of the compression-moulded PA11/rCF laminates are summarised in Table 2. Similar to the tensile results, the longitudinal compression modulus increased strongly with fibre mass content. The PA11/rCF 50 wt.% material exhibited a compression modulus of 30.20 ± 0.62 GPa, while the PA11/rCF 70 wt.% material reached 53.88 ± 2.02 GPa, corresponding to an increase of approximately 78%.

**Figure 7:**
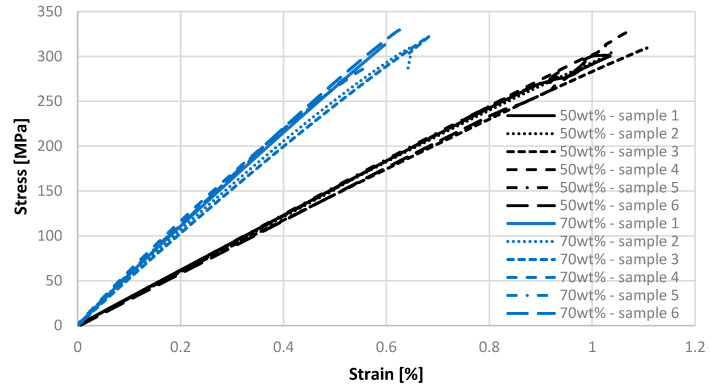
Longitudinal compressive stress–strain curves (UD 0°) of PA11/rCF 50 wt.% (*n* = 6) and 70 wt.% (*n* = 6) laminates.

Figure 7 shows the compressive stress–strain response of the UD 0° laminates. The substantial increase in compression modulus with increasing fibre content is consistent with the higher fraction of load-bearing carbon fibres and the improved fibre alignment within the laminate architecture (see Section 3.7).

Despite the substantial increase in compression modulus, the longitudinal compression strength remained nearly unchanged. The 50 wt.% material reached a compression strength of 301.2 ± 18.0 MPa, while the 70 wt.% material reached 308.0 ± 17.7 MPa. This indicates that compression strength is not governed by fibre content alone, but is also influenced by fibre waviness, local misalignment, voids, fibre-rich regions and the initiation of kink-band or microbuckling failure mechanisms. It should be noted that, while the dense fibre network at 70 wt.% improves compressive stiffness, the reduction in resin-rich regions (Figure 4b,d) increases sensitivity to localised microbuckling and defects. Consequently, the gain from higher fibre volume fraction is offset by these structural perturbations, pinning the compressive strength at approximately 300 MPa.

**Figure 8:**
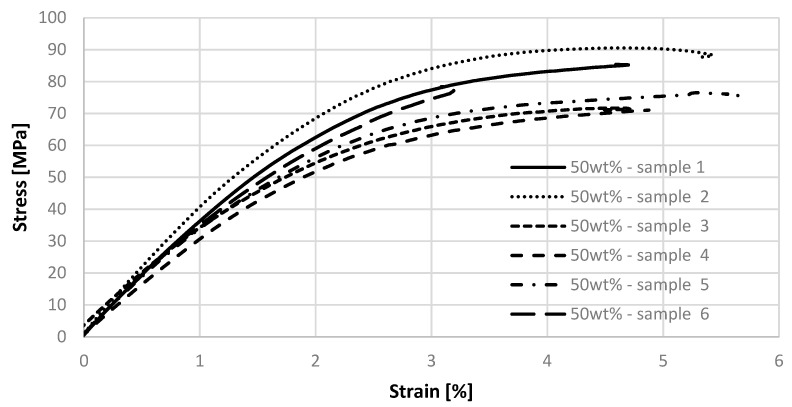
Transverse compressive stress–strain curves (UD 90°) of PA11/rCF 50 wt.% (*n* = 6) laminates.

The transverse compression behaviour is shown in Figure 8. Compression data in the UD 90° direction were only available for the PA11/rCF 50 wt.% material, as no transverse compression laminate was manufactured for the 70 wt.% configuration due to material limitations.

For the PA11/rCF 50 wt.% material, the transverse compression modulus and strength were 3.68 ± 0.38 GPa and 79.1 ± 7.7 MPa, respectively. As observed in the tensile tests, the transverse response was dominated primarily by the matrix, fibre–matrix interface quality, and local defects rather than by the axial stiffness of the carbon fibres.

### 3.5. In-Plane Shear Properties (ISO 14129)

The in-plane shear properties determined using the ±45° tensile test method are listed in Table 3. Increasing the recycled carbon fibre content resulted in a substantial improvement in both shear stiffness and shear strength. The shear modulus increased from 1.72 ± 0.21 GPa for PA11/rCF 50 wt.% material to 3.56 ± 0.58 GPa for the PA11/rCF 70 wt.% material. Similarly, the maximum shear stress increased from 75.3 ± 4.1 MPa to 116.5 ± 0.7 MPa.

The high in-plane shear modulus (G_12_ = 3.56 GPa) is a direct consequence of the mesoscopic fibre misalignment inherent to the textile tape production process, which is shown quantitatively in the following section. In accordance with laminate theory, the fraction of off-axis fibre segments deviating beyond ±10° acts as a multidirectional reinforcement network that significantly boosts shear stiffness.

**Figure 9:**
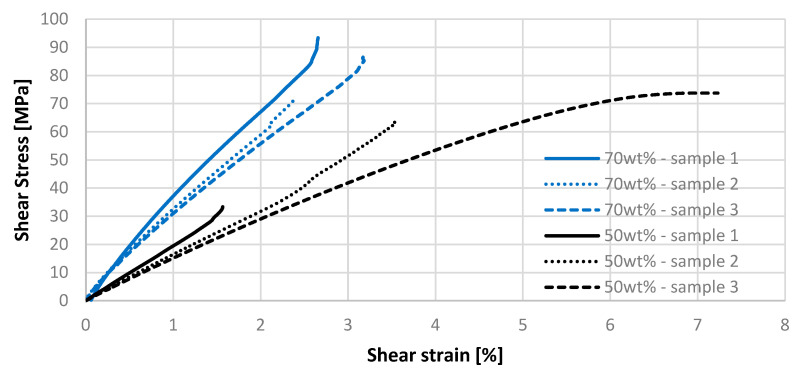
In-plane shear stress–strain curves of PA11/rCF 50 wt.% (*n* = 3) and 70 wt.% (*n* = 3) laminates.

Figure 9 shows the in-plane shear stress–strain response of the investigated laminates. The higher-fibre-content material exhibited a steeper initial slope and higher maximum shear stress, confirming the beneficial effect of increased fibre content on the shear performance of the laminate.

The shear strain at maximum stress decreased from 4.08 ± 2.83% for PA11/rCF 50 wt.% to 2.74 ± 0.40% for PA11/rCF 70 wt.%. This behaviour indicates that the higher-fibre-content laminate was not only stiffer and stronger under shear loading but also less deformable. The comparatively large scatter in shear strain observed for the 50 wt.% material suggests a more variable damage evolution, which may be associated with local variations in fibre orientation, matrix-rich regions, tape overlaps and local consolidation quality.

It should be noted that specimen counts were selected in accordance with the testing standards (ISO 527-5, ISO 14126, and ISO 14129). While the inherent heterogeneity of recycled carbon fibre (rCF) composites generally requires larger sample sizes during industrial characterisation to map material variability with high confidence probabilities, the low scatter observed in this study justifies the current sample sizes. The coefficients of variation (CV) remain below 4% for longitudinal stiffness and between 5% and 10% for matrix-dominated transverse and shear properties, which is fully comparable to the variability found in conventional virgin fibre thermoplastic laminates. This demonstrates the proper processing uniformity achieved by the textile-based tape manufacturing route.

### 3.6. Fibre Orientation Analysis

**Figure 10:**
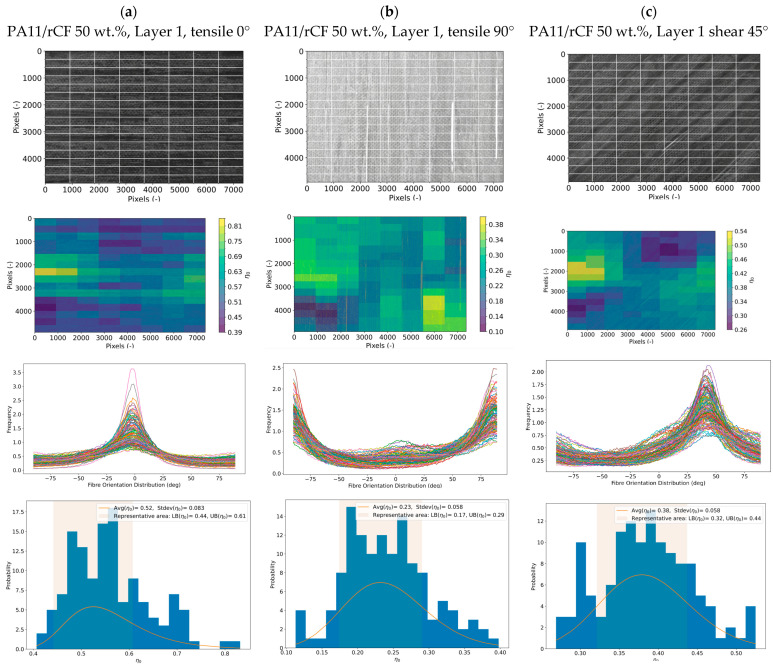
Image analysis results of the first layer of the plates: (**a**) PA11/rCF 50 wt.%, tensile 0°; (**b**) PA11/rCF 50 wt.%, tensile 90°; and (**c**) PA11/rCF 50 wt.%, shear 45°. For each configuration: the first row shows the raw acquired image of the tape layer; the second row shows the spatial distribution of the alignment coefficient η_0_ derived from the structure tensor analysis for each image segment (colour scale from 0.39 to 0.81); the third row shows the fibre orientation distribution (frequency vs. orientation angle, −90° to 90°) for each analysed segment; and the fourth row shows resulting alignment coefficient (η) distribution for different segments.

**Figure 11:**
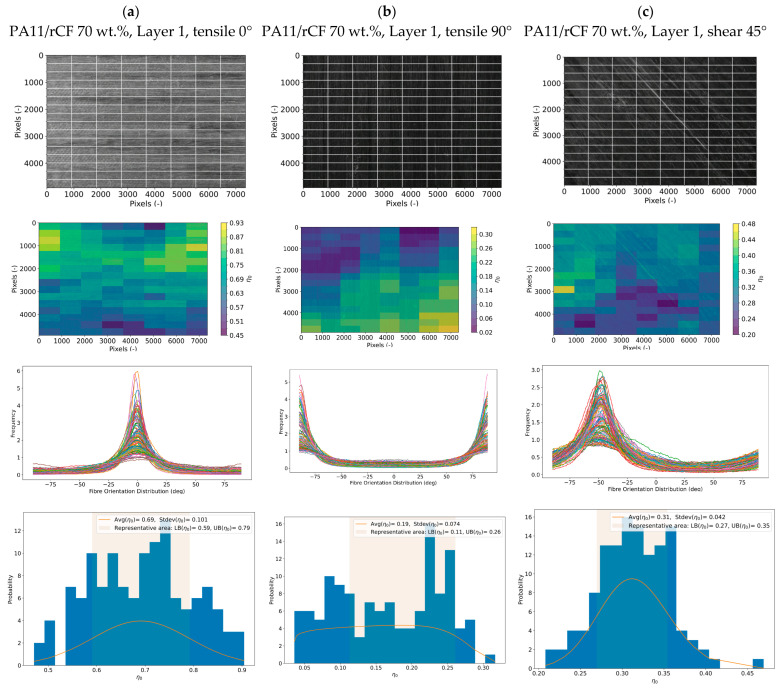
Image analysis results of the first layer of the plates: (**a**) PA11/rCF 70 wt.%, tensile 0°; (**b**) PA11/rCF 70 wt.%, tensile 90°; and (**c**) PA11/rCF 70 wt.%, shear 45°. Row layout and colour scale as described for Figure 10.

The 2D mesoscopic image analysis confirmed that the carding- and drafting-based tape manufacturing process produced a distinct preferential fibre orientation. Representative analysis results for the first layer of the PA11/rCF 50 wt.% and 70 wt.% materials are shown in Figure 10 and Figure 11, respectively. In all laminate configurations, the structure tensor analysis revealed a dominant fibre orientation aligned with the tape manufacturing direction, indicating that the textile processing route effectively aligned the discontinuous recycled carbon fibres.

The orientation maps further revealed local variations in alignment quality. In addition to highly aligned fibre regions, areas with broader orientation distributions and locally fuzzy fibre arrangements were observed. Such features are typical of aligned staple fibre architectures and may originate from fibre opening, carding, drafting, tape handling, and laminate assembly. Quantitative evaluation of the fibre orientation distributions showed that approximately 44.0 ± 7.1% of the analysed fibre regions were aligned within ±5° of the dominant fibre direction and 68.8 ± 8.3% within ±10°. These values indicate a pronounced preferential fibre orientation while also highlighting the presence of local misalignment and orientation scatter within the laminate structure.

**Figure 12:**
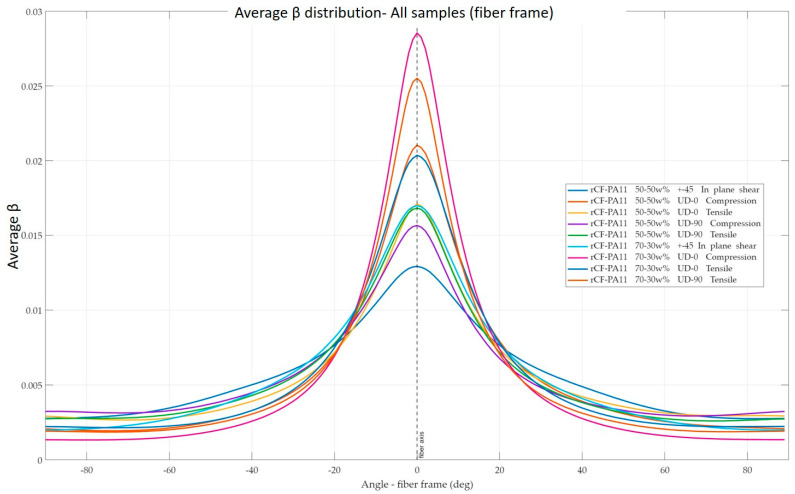
Orientation histograms averaged over all layers of the investigated laminates.

The averaged angular probability density distributions further confirmed the highly anisotropic fibre orientation state generated by the carding and drafting process (Figure 12). All investigated laminates exhibited a pronounced peak at 0° in the fibre reference frame, corresponding to the dominant alignment direction introduced during tape manufacturing. The distributions consisted of a narrow central orientation peak combined with broader tails towards ±90°, indicating a highly aligned main fibre population together with a smaller fraction of locally misaligned fibres and fuzzy tape-edge regions.

The PA11/rCF 70 wt.% laminates generally exhibited sharper and higher orientation peaks compared with the PA11/rCF 50 wt.% material. In particular, the UD 0° compression laminates of the 70 wt.% material showed the highest central probability density, indicating the strongest local alignment and lowest angular scatter. In contrast, the ±45° shear laminates and UD 90° configurations showed broader distributions and reduced peak amplitudes, reflecting the increased sensitivity of these layups to local fibre steering, tape overlap and re-orientation effects during laminate assembly.

**Figure 13:**
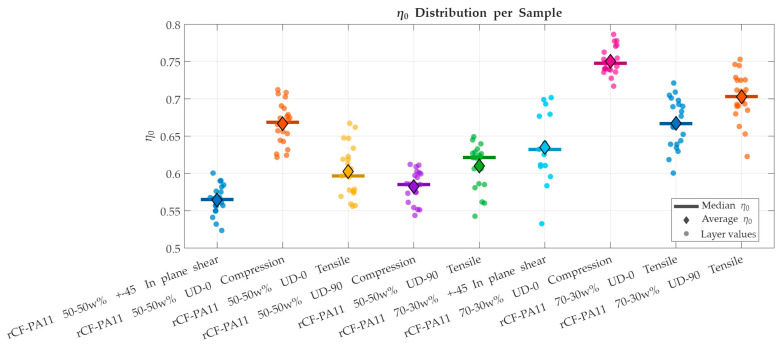
Alignment coefficient *η*_0_ derived from virtually stacked optical images acquired ply by ply for all laminates (alignment coefficient definition is given in Equation (1)).

The calculated alignment coefficient η_0_ also showed clear differences between the investigated laminate configurations (Figure 13). The lowest η_0_ values were observed for the PA11/rCF 50 wt.% ±45° shear laminates, with average values around η0 ≈ 0.56, indicating a broader orientation distribution and increased local angular deviation. The PA11/rCF 50 wt.% UD 0° compression laminates exhibited significantly higher alignment values of approximately η_0_ ≈ 0.67, whereas the PA11/rCF 70 wt.% UD 0° compression laminates reached the highest values with η_0_ ≈ 0.75.

The image analysis is particularly valuable for interpreting the measured mechanical behaviour. Although the surface-based structure tensor approach provides a conservative estimate of fibre alignment compared with three-dimensional techniques, it remains a sensitive indicator of process-induced variations in fibre architecture [1,11].

Higher η_0_ values corresponded to increased longitudinal tensile and compression stiffness, consistent with improved load transfer efficiency along the fibre direction. The PA11/rCF 70 wt.% laminates combined the highest η_0_ values with the highest longitudinal tensile modulus (48.14 ± 4.55 GPa) and compression modulus (53.88 ± 2.02 GPa). In contrast, configurations with broader orientation distributions and lower η_0_ values showed lower stiffness and increased mechanical scatter. This trend is particularly relevant for compression loading, where local fibre waviness and angular deviations promote premature microbuckling and kink-band initiation.

The boxplot analysis in Figure 13 additionally revealed that the 70 wt.% laminates showed lower scatter of η_0_ in the UD 0° compression configuration compared with the 50 wt.% materials, indicating a more homogeneous fibre architecture. Nevertheless, all configurations still exhibited local deviations and outliers, confirming that the discontinuous fibre architecture remains sensitive to fibre transport, drafting stability and tape handling during layup. These local orientation variations may influence the mechanical response of the laminates, particularly under compression loading where fibre misalignment can promote premature microbuckling and kink-band formation.

Overall, the results are consistent with previous studies on aligned discontinuous carbon fibre composites [1,11] and demonstrate that mesoscopic fibre orientation is a key parameter governing the anisotropic behaviour of aligned rCF laminates as well as its appropriate processing conditions [12].

### 3.7. SEM Fracture Surface Analysis

**Figure 14:**
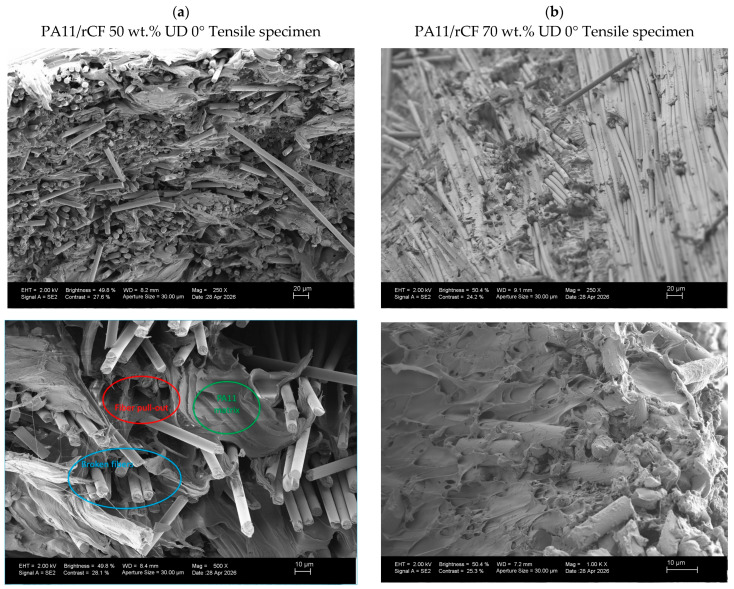
SEM micrographs of fracture surfaces from UD 0° tensile specimens: (**a**) PA11/rCF 50 wt.% laminate; (**b**) PA11/rCF 70 wt.% laminate.

Representative SEM micrographs of fracture surfaces from UD 0° tensile specimens are shown in Figure 14. Both the PA11/rCF 50 wt.% laminate (Figure 14a) and the PA11/rCF 70 wt.% laminate (Figure 14b) exhibited exposed carbon fibres, fibre pull-out, local matrix coverage, and regions with limited impregnation.

The PA11/rCF 70 wt.% laminate generally exhibited a higher density of exposed fibres, reflecting its increased fibre content, whereas the PA11/rCF 50 wt.% laminate showed more extensive matrix-rich regions. In both materials, local variations in matrix coverage and fibre distribution were observed, indicating that the fracture behaviour was influenced by microstructural heterogeneities originating from tape manufacturing and laminate consolidation.

The observed fracture features are consistent with the mechanical test results and suggest that local fibre–matrix interaction, impregnation quality, and residual defects play an important role in determining the tensile and compression performance of the laminates. In particular, regions with limited matrix coverage or insufficient consolidation may act as local stress concentrators and promote premature damage initiation.

### 3.8. Fibre Volume Content

The fibre volume contents (FVCs) determined using thermogravimetric analysis (TGA), muffle furnace decomposition, and chemical dissolution are summarised in Table 4. All three methods consistently indicated a higher fibre content for the PA11/rCF 70 wt.% material compared with the PA11/rCF 50 wt.% material. However, substantial differences were observed between the thermal and chemical methods.

Note that fibre volume contents (FVC) reported in this table were calculated using Equation (3), based on a carbon fibre density of 1.80 g·cm^−3^ and a PA11 matrix density of 1.03 g·cm^−3^.

**Figure 15:**
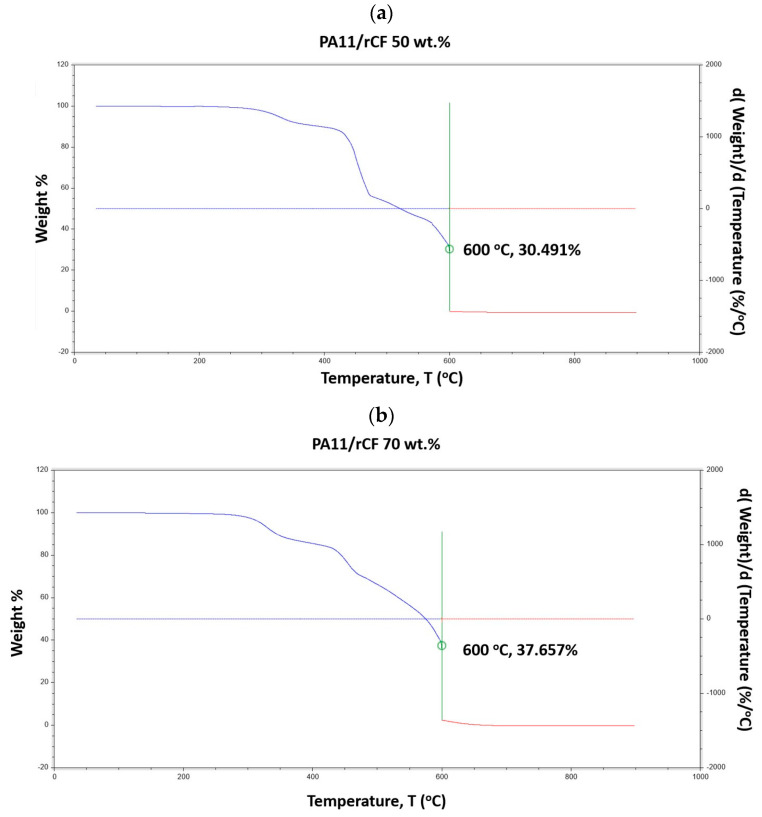
Thermogravimetric analysis (TGA) and derivative thermogravimetric (DTG) curves of the PA11/rCF composites: (**a**) PA11/rCF 50 wt.%; (**b**) PA11/rCF 70 wt.%.

The TGA curves of the PA11/rCF composites (Figure 15a,b) exhibit a single-step thermal decomposition of the PA11 matrix occurring between 300 and 500 °C. The residual masses recorded at 600 °C, which correspond to the carbon fibre fraction, were found to be 30.49 wt.% and 37.66 wt.% for the PA11/rCF 50 wt.% and 70 wt.% composites, respectively. The corresponding DTG curves display a single dominant degradation peak, confirming that no additional decomposition process takes place within the matrix phase. The fibre content values derived from these curves were used to calculate the TGA-based FVC values reported in Table 4.

The TGA and muffle furnace methods yielded very similar results for both material systems, indicating good agreement between the two thermal decomposition techniques. For the PA11/rCF 50 wt.% material, fibre volume contents of 19.97 vol.% and 18.3 vol.% were obtained using TGA and muffle furnace decomposition, respectively. Similarly, for the PA11/rCF 70 wt.% material, both methods resulted in nearly identical values of 25.88 vol.% and 25.4 vol.%.

In contrast, the chemical dissolution method produced significantly higher fibre volume contents of 34.15 vol.% for the PA11/rCF 50 wt.% material and 56.72 vol.% for the PA11/rCF 70 wt.% material. These values are considerably more consistent with the nominal fibre mass fractions used during tape production and with the mechanical performance of the laminates. In particular, the longitudinal tensile modulus of 48.14 GPa and compression modulus of 53.88 GPa obtained for the PA11/rCF 70 wt.% laminates would be difficult to achieve at a fibre volume content below 30 vol.%, suggesting that the chemical dissolution results provide a more realistic representation of the actual laminate composition.

The discrepancy between the thermal and chemical methods is attributed primarily to partial fibre degradation during high-temperature analysis. Although carbon fibres are generally thermally stable, oxidation during the air-burning stage of TGA or prolonged exposure in the muffle furnace can lead to mass losses that artificially reduce the calculated fibre content. Furthermore, fibre surface oxidation, residual ash formation, and uncertainties in the thermal decomposition behaviour of the matrix may contribute to inaccuracies in the thermal methods. The chemical dissolution method avoids these limitations by selectively removing the PA11 matrix while preserving the carbon fibres. Consequently, it is considered the most reliable approach for determining the fibre mass and fibre volume content of the investigated PA11/rCF composites. The resulting fibre volume contents of 34.15 vol.% and 56.72 vol.% are also in good agreement with the observed increase in stiffness and strength between the two material systems. Furthermore, the fibre volume contents obtained through chemical dissolution are in close agreement with the theoretical of 36.4 vol.% and 57.2 vol.% calculated from the nominal fibre mass fractions of 50 wt.% and 70 wt.% rCF, respectively.

Overall, the results demonstrate that the fibre content was successfully increased from approximately 34 vol.% to 57 vol.% through modification of the tape composition. This increase is consistent with the substantial improvements observed in tensile, compression, and shear stiffness and confirms that fibre content is one of the dominant parameters governing the mechanical performance of aligned recycled carbon fibre thermoplastic composites. While chemical dissolution is considered the most reliable of the three methods applied in this study, for the reasons discussed above, we acknowledge that a fully independent, bulk-scale validation of fibre volume content, for example via buoyancy/density-based determination according to ISO 1183-1:2025 [24], or FTIR/Raman spectroscopy of the dissolved fibre residue to confirm complete matrix removal, was not performed in this study and is recommended as a valuable direction for future work on this material system. Such methods would provide additional confirmation independent of both the thermal and dissolution-based approaches used here, particularly for recycled fibre systems where established protocols for continuous virgin fibre composites may not directly apply.

## 4. Discussion

The present study demonstrates that highly aligned recycled carbon fibre (rCF) tapes combined with a bio-based PA11 matrix can be transformed into structural composite laminates exhibiting mechanical properties comparable to those reported for aligned discontinuous fibre composites manufactured from virgin fibres. The combination of a textile-based alignment process, secondary tape consolidation, and compression moulding enabled the production of laminates with fibre volume contents up to 56.7 vol.% and pronounced preferential fibre orientation.

### 4.1. Influence of Fibre Volume Content on Mechanical Performance

The chemical dissolution measurements revealed fibre volume contents of 34.15 vol.% and 56.72 vol.% for the PA11/rCF 50 wt.% and 70 wt.% materials, respectively. These values are substantially higher than those obtained using TGA and muffle furnace decomposition and are considered the most representative values for the actual laminate architecture. The significantly higher FVC values are also consistent with the measured tensile, compression and shear properties and therefore provide a more realistic basis for mechanical interpretation.

Increasing the fibre volume content from 34.15 vol.% to 56.72 vol.% resulted in a substantial increase in stiffness under all loading conditions. The longitudinal tensile modulus increased by approximately 48%, from 32.57 GPa to 48.14 GPa, while the longitudinal compression modulus increased by approximately 78%, from 30.20 GPa to 53.88 GPa. Similarly, the in-plane shear modulus more than doubled from 1.72 GPa to 3.56 GPa. These improvements demonstrate that the carding and tape-forming process successfully transferred the intrinsic stiffness of the recycled carbon fibres into the final laminate architecture.

The increase in tensile strength from 718.9 MPa to 866.2 MPa is less pronounced than the increase in stiffness. This indicates that while increasing fibre content effectively increases the load-bearing capacity of the laminate, failure remains controlled by local defects such as fibre misalignment, fibre bundle discontinuities, voids and imperfect impregnation. Similar observations have been reported for aligned discontinuous fibre composites, where strength is considerably more sensitive to processing defects than stiffness.

To assess whether the observed differences between the 50 wt.% and 70 wt.% material properties (Table 1, Table 2 and Table 3) are statistically meaningful, a two-sample Welch’s *t*-test [25,26] was performed for each measured property (Table 5).

In Table 5, the t-value reflects the size of the difference between the two group means relative to their combined scatter; the degrees of freedom (df), calculated using the Welch–Satterthwaite equation [26], account for unequal variance and sample size between groups; and the *p*-value gives the probability of observing a difference this large if no true difference existed, with *p* < 0.05 taken as statistically significant [26]. The results confirm that the increases in longitudinal and transverse tensile modulus, tensile strain at failure, compression modulus, and shear modulus and strength are all statistically significant (*p* < 0.05). Four properties did not reach statistical significance: transverse tensile strength (*p* = 0.466) and shear strain at maximum stress (*p* = 0.499), both matrix- and interface-dominated properties not expected to scale strongly with fibre content; longitudinal tensile strength, which was borderline (*p* = 0.059); and longitudinal compression strength (*p* = 0.524), despite the substantial increase in compression modulus over the same fibre content range. The authors consider this last result a mechanistic effect rather than a statistical artefact, as discussed in Section 4.3, Section 4.4 and Section 4.5. We will discuss that compression strength in these laminates appears to be governed by a distinct failure mechanism, namely defect- and alignment-driven microbuckling, that does not scale with fibre content in the same way as stiffness.

### 4.2. Relationship Between Fibre Alignment and Mechanical Properties

The image analysis revealed a pronounced preferential fibre orientation generated by the carding and drafting process. Approximately 44.0 ± 7.1% of all analysed fibre regions were aligned within ±5° of the dominant fibre direction, while 68.8 ± 8.3% were aligned within ±10°. These values are comparable to those reported by Detzel et al. [13] and Zweifel et al. [11] for aligned recycled carbon staple fibre systems and confirm the effectiveness of the textile alignment process.

The orientation histograms exhibited a sharp peak around 0°, confirming that most fibres contributed effectively to load transfer in the longitudinal direction. The PA11/rCF 70 wt.% laminates showed consistently sharper peaks and higher alignment coefficients than the corresponding 50 wt.% material, indicating improved mesoscopic fibre alignment.

The derived alignment coefficient η_0_ exhibited a clear correlation with the measured stiffness values. The highest η_0_ values were observed for the PA11/rCF 70 wt.% compression laminates (η_0_ ≈ 0.75), which also exhibited the highest tensile and compression moduli. In contrast, the ±45° shear laminates showed the lowest η_0_ values (η_0_ ≈ 0.56), reflecting broader fibre orientation distributions and lower effective reinforcement efficiency.

These results confirm that fibre alignment is a critical parameter controlling stiffness development in aligned recycled fibre composites. Even relatively small angular deviations can significantly reduce the effective longitudinal modulus and promote local stress concentrations. The structure tensor analysis therefore provides a valuable process quality metric that links manufacturing quality directly to mechanical performance.

### 4.3. Compression Behaviour and Defect Sensitivity

While tensile stiffness and strength increased with fibre volume content, the compression strength remained nearly constant at approximately 300 MPa despite the substantial increase in fibre content. The PA11/rCF 50 wt.% and 70 wt.% laminates exhibited compressive strengths of 301 MPa and 308 MPa, respectively.

This behaviour indicates that compressive failure is not governed primarily by fibre content but rather by local imperfections within the fibre architecture. Compression loading is particularly sensitive to fibre waviness, local misalignment, resin-rich regions and voids, which can trigger premature microbuckling and kink-band formation. The image analysis supports this interpretation because even the most highly aligned laminates still exhibited measurable local orientation scatter and outliers.

Consequently, future improvements in compressive performance will likely require optimisation of fibre transport during carding, improved control of tape thickness uniformity and further reduction in local fibre waviness rather than simply increasing fibre content.

### 4.4. Influence of Consolidation Quality

The compression moulding trials highlighted the importance of consolidation quality for highly filled thermoplastic tapes. Whereas the PA11/rCF 50 wt.% material could be consolidated using the standard process cycle, the PA11/rCF 70 wt.% material required multiple re-consolidation cycles at increasing pressure levels up to 100 bar to achieve acceptable impregnation quality.

Cross-sectional microscopy revealed that insufficient pressure led to locally dry regions and incomplete matrix infiltration. Even after secondary consolidation, isolated voids and fibre-rich areas remained visible within the laminate structure. These features are expected to reduce transverse tensile strength, promote interfacial debonding and act as initiation sites for compressive failure.

The nearly unchanged transverse tensile strength despite a substantial increase in fibre content further supports this interpretation. Both material systems exhibited transverse strengths close to 50 MPa, indicating that transverse behaviour is controlled primarily by matrix continuity and fibre–matrix adhesion rather than fibre volume fraction.

### 4.5. Fracture Mechanisms

SEM observations provide further evidence for the dominant failure mechanisms. Fracture surfaces revealed extensive fibre pull-out, exposed fibre bundles and locally insufficient matrix coverage. These observations indicate that although load transfer in the fibre direction is efficient, interfacial debonding and incomplete impregnation still contribute significantly to failure.

The presence of fibre pull-out rather than exclusively fibre fracture suggests that the fibre–matrix interface remains a limiting factor for strength development. This behaviour is commonly observed in recycled carbon fibre composites due to the absence or degradation of sizing during fibre reclamation. Consequently, additional improvements may be achievable through fibre surface treatments, optimised sizing systems or tailored coupling agents compatible with PA11.

### 4.6. Implications for Sustainable Structural Composites

From a sustainability perspective, the achieved combination of high fibre alignment, fibre volume contents up to 56.7 vol.% and tensile strengths approaching 900 MPa demonstrates the considerable potential of recycled carbon fibres for structural thermoplastic applications. The measured properties significantly exceed those typically reported for commercially standard, randomly oriented short-fibre compounds [27]. Furthermore, they successfully bridge the performance gap to high-end materials, approaching the lower boundary of continuous virgin carbon fibre and polyamide 12 (vCF/PA12) industrial tapes. To contextualise the mechanical performance of the developed PA11/rCF tapes, they are compared with a commercially available continuous vCF/PA12 tape [17]. The vCF/PA12 reference exhibits a longitudinal Young’s modulus of approximately 132.6 GPa, a transverse modulus of 6.6 GPa, a shear modulus of 2.0 GPa, and a fibre volume content of 57.3%. In comparison, the PA11/rCF 70 wt.% tape developed in this study achieves a longitudinal modulus of 48.14 ± 4.55 GPa, a transverse modulus of 3.91 ± 0.21 GPa, and a shear modulus of 3.56 ± 0.58 GPa, at a comparable fibre volume content of 56.72%. The PA11/rCF 50 wt.% tape shows a longitudinal modulus of 32.57 ± 0.55 GPa, a transverse modulus of 2.92 ± 0.18 GPa, and a shear modulus of 1.72 ± 0.21 GPa, at a fibre volume content of 34.15%. It should be noted that the recycled carbon fibres used in this study originate from T700SC production offcuts, a standard-modulus, high-strength fibre grade, whereas the vCF/PA12 reference is based on a higher modulus continuous fibre (IM7/T800-class). Part of the lower longitudinal stiffness observed for the PA11/rCF tapes is therefore attributable to the intrinsically lower modulus of the T700SC fibre itself, in addition to the discontinuous fibre architecture. Notably, the in-plane shear modulus of the PA11/rCF 70 wt.% tape exceeds that of the vCF/PA12 reference, which is attributed to the mesoscopic off-axis fibre fraction inherent to the textile-based alignment process. These results indicate that, despite relying on a lower-modulus, discontinuous recycled fibre, the developed tapes achieve a substantial fraction of the stiffness of continuous virgin fibre tapes.

The results demonstrate that textile-based alignment technologies can successfully bridge the gap between low-value recycled fibre compounds and high-performance structural composites. Combined with the renewable origin of PA11, this approach provides a promising route toward lightweight composite structures with significantly reduced environmental impact while maintaining engineering-level mechanical performance.

## 5. Conclusions

This study demonstrated the feasibility of manufacturing highly aligned recycled carbon fibre (rCF)-reinforced composite laminates based on bio-based polyamide 11 (PA11) using a textile-based tape production route followed by compression moulding. The developed process successfully transformed discontinuous recycled carbon fibre staples into structurally relevant composite tapes with high fibre alignment and mechanical performance.

1. The combination of carding, drafting, tape formation and compression moulding produced highly anisotropic laminates with tensile moduli up to 48.14 ± 4.55 GPa and tensile strengths up to 866.2 ± 127.2 MPa. These values demonstrate that a significant proportion of the intrinsic mechanical potential of recycled carbon fibres can be recovered through alignment and consolidation.
2. Chemical dissolution measurements revealed fibre volume contents of 34.15 vol.% and 56.72 vol.% for the PA11/rCF 50 wt.% and 70 wt.% materials, respectively. The increase in fibre volume fraction resulted in substantial improvements in longitudinal tensile, compression and shear stiffness, confirming the effectiveness of the tape manufacturing process in producing highly loaded structural laminates.
3. Structure tensor analysis demonstrated that approximately 68.8 ± 8.3% of the analysed fibre regions were aligned within ±10° of the dominant fibre direction. The higher-fibre-content laminates exhibited both higher alignment coefficients and higher mechanical properties, confirming the strong relationship between fibre orientation quality and laminate stiffness. The image analysis methodology proved to be an effective process quality tool for evaluating aligned recycled fibre architectures.
4. Although the longitudinal compression modulus increased from 30.20 ± 0.62 GPa to 53.88 ± 2.02 GPa, the compressive strength remained approximately constant at around 300 MPa. This indicates that compressive failure is governed primarily by local fibre waviness, misalignment, voids and incomplete impregnation rather than by fibre content alone.
5. The PA11/rCF 70 wt.% tapes required multiple consolidation cycles at pressures up to 100 bar to achieve acceptable laminate quality. Cross-sectional microscopy and SEM analysis revealed locally dry regions, fibre-rich zones and interfacial defects that are likely responsible for the limited improvements in strength compared with stiffness.
6. The achieved fibre volume contents, alignment quality and mechanical properties demonstrate that recycled carbon fibres can be converted into high-value semi-finished products suitable for lightweight engineering applications. The developed tape architecture offers a sustainable alternative to conventional virgin fibre thermoplastic composites while maintaining mechanical properties relevant for structural components.

Overall, the results demonstrate that textile-based alignment technologies provide an effective route for upgrading recycled carbon fibres into high-performance thermoplastic composites. Future work should focus on improving fibre–matrix interfacial bonding, reducing void content through optimised consolidation strategies, investigating fatigue and impact behaviour, and scaling the tape manufacturing process towards continuous industrial production. Furthermore, the integration of advanced quality control methods, such as automated fibre orientation monitoring and quantitative void analysis, will be essential for achieving the consistency required for demanding structural applications.

## Figures and Tables

**Table 1:** Tensile properties of compression-moulded PA11/rCF plates according to ISO 527-5.

Material	Orientation	*n*	E_1_, E_2_ [GPa]	σ_m_ [MPa]	ε_m_ [%]
PA11/rCF 50 wt.%	UD 0°	5	32.57 ± 0.55	718.9 ± 34.2	2.17 ± 0.11
PA11/rCF 70 wt.%	UD 0°	5	48.14 ± 4.55	866.2 ± 127.2	1.78 ± 0.18
PA11/rCF 50 wt.%	UD 90°	3	2.92 ± 0.18	50.6 ± 4.3	2.29 ± 0.50
PA11/rCF 70 wt.%	UD 90°	3	3.91 ± 0.21	52.9 ± 2.0	2.45 ± 0.43

**Table 2:** Compression properties of compression-moulded PA11/rCF laminates determined according to ISO 14126.

Material	Orientation	*n*	E_c_ [GPa]	σ_cm_ [MPa]
PA11/rCF 50 wt.%	UD 0°	6	30.20 ± 0.62	301.2 ± 18.0
PA11/rCF 70 wt.%	UD 0°	6	53.88 ± 2.02	308.0 ± 17.7
PA11/rCF 50 wt.%	UD 90°	6	3.68 ± 0.38	79.1 ± 7.7

**Table 3:** In-plane shear properties of compression-moulded PA11/rCF plates according to ISO 14129.

Material	*n*	G_12_ [GPa]	τ_12m_ [MPa]	γ_m_ [%]
PA11 rCF 50 wt.%	3	1.72 ± 0.21	75.3 ± 4.1	4.08 ± 2.83
PA11 rCF 70 wt.%	3	3.56 ± 0.58	116.5 ± 0.7	2.74 ± 0.40

**Table 4:** Fibre volume content determined through thermogravimetric analysis, muffle furnace decomposition and chemical dissolution.

Specimen	FVC Through TGA [vol.%]	FVC Through Muffle Furnace [vol.%]	FVC Chemical Dissolution [vol.%]
PA11/rCF 50 wt.%	19.97	18.3	34.15
PA11/rCF 70 wt.%	25.88	25.4	56.72

**Table 5:** Statistical significance of property differences between PA11/rCF 50 wt.% and 70 wt.% laminates (two-sample Welch’s *t*-test, α = 0.05; see Table 1, Table 2 and Table 3 for the underlying mean ± SD and *n*).

Property	t	df	*p*-Value	Significant (*p* < 0.05)
Tensile modulus E_1_ (UD 0°)	7.60	4.1	0.0014	Yes
Tensile strength σ_m_ (UD 0°)	2.50	4.6	0.059	No
Tensile strain ε_m_ (UD 0°)	−4.13	6.6	0.0049	Yes
Transverse tensile modulus E_2_ (UD 90°)	6.20	3.9	0.0037	Yes
Transverse tensile strength (UD 90°)	0.84	2.8	0.466	No
Compression modulus E_c_	27.45	5.9	<0.0001	Yes
Compression strength σ_cm_	0.66	10.0	0.524	No
Shear modulus G_12_	5.17	2.5	0.021	Yes
Shear strength τ_12m_	17.16	2.1	0.0026	Yes
Shear strain γ_m_	−0.81	2.1	0.499	No

## Data Availability

All data can be extracted from the figures and tables. The data presented in this study are available on request from the corresponding author.
